# Gallic acid protects the liver against NAFLD induced by dust exposure and high-fat diet through inhibiting oxidative stress and repressing the inflammatory signaling pathways NF-kβ/TNF-α/IL-6 in Wistar rats

**DOI:** 10.22038/AJP.2021.17835

**Published:** 2021

**Authors:** Hafseh Fanaei, Seyyed Ali Mard, Alireza Sarkaki, Gholamreza Goudarzi, Layasadat Khorsandi

**Affiliations:** 1 *Persian Gulf Physiology Research Center, Medical Basic Sciences Research Institute, Ahvaz Jundishapur University of Medical Sciences, Ahvaz, Iran *; 2 *Air Pollution and Respiratory Diseases Research Center, Ahvaz Jundishapur University of Medical Sciences, Ahvaz, Iran*; 3 *Environmental Technologies Research Center, Ahvaz Jundishapur University of Medical Sciences, Ahvaz, Iran *; 4 *Cellular and Molecular Research Center, Medical Basic Sciences Research Institute, Department of Anatomical Sciences, School of Medicine, Ahvaz Jundishapur University of Medical Sciences, Iran*

**Keywords:** High fat diet, Dust, NAFLD, Gallic acid, ROS, TNF-α, Rat

## Abstract

**Objective:**

The burden of diseases and death related to environmental pollution is becoming a major public health challenge. This study was designed to evaluate the deleterious effects of a combination of dust exposure and high-fat diet on liver function. Gallic acid as a potent antioxidant was used to prevent/alleviate non-alcoholic fatty liver disease (NAFLD) in rats exposed to dust and HFD.

**Materials and Methods:**

24 rats were randomly divided into 3 experimental groups: HFD+Clean air, HFD+N/S+Dust and HFD+gallic acid+Dust. Animals were exposed to CA/ dust for six weeks on alternate days. At the end of the experiments, rats were anesthetized and samples were taken to perform molecular, biomedical, and histopathological evaluations.

**Results:**

Dust exposure induced NAFLD features in rats under HFD. Dust exposure and HFD disrupted liver enzymes and lipid profile. Dust exposure and HFD increased liver MDA level, mRNA expression of *NF-Kβ*, *TNF-α*, *IL-6*, *Nrf2*, *HO1* and miRs122, and 34a. Dust+HFD also decreased liver total antioxidant capacity level. Pretreatment with GA improved almost studied variables in the HFD+GA+Dust group.

**Conclusion:**

The present study showed that HFD given for 6 weeks and dust exposure induced NAFLD in Wistar rats through inducing oxidative stress. Oxidative stress through activating the inflammatory pathways caused NAFLD features. GA pretreatment by inhibiting oxidative stress, effectively protected liver functions against HFD+Dust induced inflammation.

## Introduction

The burden of diseases and death related to environmental pollution is becoming a major public health challenge, especially in developing countries. Gaseous pollutants (NOx, ozone, sulfur dioxide and carbon monoxide), persistent organic pollutants (dioxins, pesticides and furans), heavy metals (mercury, silver, lead, nickel, vanadium, manganese chromium and cadmium), and particulate matters (PMs) constitute air pollution compounds (Kampa and Castanas, 2008 ▶).

Particulate matter (PM) is a generic term used for various kinds of air pollutant, with different sizes and compositions, that are produced by natural and anthropogenic phonemes (Poschl, 2005 ▶). The size of the particle matters according to different categories, was defined as: Ultrafine, Fine and Coarse particles(aerodynamic diameter smaller than 0.1 µm, equal to 1 µm and larger than 1 µm, respectively) (Kampa and Castanas, 2008 ▶).

 The existing literatures on air pollution have mostly focused on particulate matters and gaseous pollutant (SO_2_, ozone and CO) and less, on the effect of dust on health status (Al-Taiar and Thalib, 2014 ▶). The natural phenomenon, dust storm, transfers soil particles to another place, sometimes miles away from the point of dust storms origin (Al-Taiar and Thalib, 2014 ▶). 

Dust storm may carry fine and coarse particle matter fractions (Zauli Sajani et al., 2011 ▶) bio-particulates and microorganisms, pollen, and related protein and lipid components (Gonzalez-Martin et al., 2014 ▶). In spite of high frequency of dust storms in the Middle East, few studies have focused on its public health effects.

A previous study showed that particle matter exposure via induction of oxidative stress, induced liver inflammation that have critical role in liver pathogenesis (Zheng et al., 2015 ▶). Mice exposed to PM2.5 demonstrated increased mRNA expressions of inflammatory mediators such as TNFα and IL-6 and showed Non-alcoholic fatty liver diseases (Tan et al., 2009 ▶). Non-alcoholic fatty liver disease is a spectrum of liver diseases ranging from simple non-alcoholic fatty liver (NAFL), to non-alcoholic steatohepatitis (NASH), and finally, liver irreversible cirrhosis (Brunt, 2001 ▶).

A previous study showed that rats with sub-chronic exposure to PM2.5, exhibited liver histopathological changes and elevated serum aspartate aminotransferase and alanine aminotransferase (Li et al., 2018 ▶). Exposure to ultrafine PMs has been indicated to increase hepatic levels of malondialdehyde which shows systemic oxidative stress, and enhance the gene expression of antioxidants related to Nrf2 (Araujo et al., 2008 ▶).

Nrf2 as a transcription factor regulates antioxidant response and promotes cellular pathways protecting against oxidative stress. Nrf2 plays vital role in almost all organs such as the liver, brain, and lung. A study demonstrated that exposure to air pollutants activated Nrf2 pathway (Jang et al., 2008 ▶).

Heme oxygenase 1 (HO1) catalyzes degradation of heme, produces biliverdin and causes a general response to ROS-induced oxidative stress (Ryter et al., 2006 ▶). A pervious study showed that exposure to PM2.5 enhances liver inflammation and causes overexpression of HO1mRNA (Li et al, 2018 ▶).

MicroRNAs (miRNAs) have important roles in physiological processes such as cell growth, differentiation and development (Bernardi et al., 2013 ▶). miR-122 shows liver health status and recognizes specific miRs in the liver (Siaj et al., 2012 ▶). MiR-34a reflects liver damage, and a direct correlation between the serum level of miR-34 and liver injury has been proven (McDaniel et al., 2014 ▶).

Gallic acid (GA) as a phenolic compound is abundant in vegetables, tea, berries and wine. GA possesses different beneficial activities, including anti-inflammatory (Hsiang et al., 2013 ▶), anti-obesity (Jang et al., 2008 ▶) and hepato protective effects (Chao et al., 2014 ▶). Thus, this study was designed to evaluate the deleterious effects of a combination of dust exposure and high fat diet, on liver function. Gallic acid as a potent antioxidant was also used to prevent/alleviate NAFLD in rats exposed to dust and high-fat diet.

## Materials and Methods

**Chemicals**

Gallic acid was purchased from sigma Aldrich Co. (Germany). Kits for measuring malondialdehyde (MDA) and total antioxidant capacity (TAC) were purchased from ZellBio Co. (Germany). Kits for determination of ALT, AST, ALP, TG, cholesterol and HDL were purchased from Pars Azmun Company (Iran).

**Animals grouping**

Twenty-four adult male Wistar rats (200-250 g) were purchased from the animal house of AJUMS. Animals were housed in the standard cage for one week before initiation of the experiment. The animals were kept in the animal house of AJUMS, Ahvaz, Iran, under a dark-light cycle of 12 hr and a temperature of 22±2^o^C. Rats had free access to standard rat chow diet and tap water.

Rats were divided randomly into 3 groups (n=8 in each): HFD+CA (rats under high fat diet, exposed to clean air), HFD+N/S+Dust (animals under HFD received normal saline as vehicle just before dust exposure), and HFD+GA+Dust (rats under HFD, received oral gallic acid at 100 mg/kg, just before dust exposure). All rats were exposed to clean air or dust for 6 weeks (three days a week on alternate days). The following materials were added to standard rats’diet to make HFD including: cholesterol (0.4%), and beef tallow (30%) and supplemented with 30% fructose (Savari et al.). Animals in none of the experimental groups had free access to food and water during dust exposure period (Niwa et al., 2008 ▶). All protocols and experiments were approved by Experimental Animals Ethics Committee of AJUMS (IR.AJUMS.ABHC.REC.1397.060).

**Dust sampling, and area of study**

Dust was collected in autumn (2018) from Ahvaz, the capital city of southwest Province of Iran, Khuzestan. This city is located at 31° 20 N, 48° 40 E geographically and is18 m above sea level (Heidari-Farsani et al., 2013 ▶). To collect dust, we placed large dishes on the Golestan medical students’dormitory roof for 3 months; then, the settled dust was collected and used in this study.

**Dust exposure**

**Figure 1:**
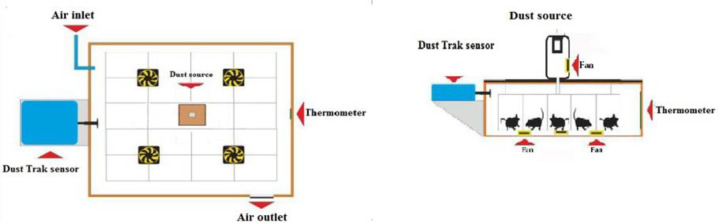
A schematic view of dust exposure cage

To create a dusty environment, dust exposure chamber was designed (Figure 1). Whole body exposure was performed 5 hr per day, 3 days per week (for 6 weeks) on alternate days, for a total of 90 hours. The concentration of PM10 during the study was 500-2000 µg/m^3^. On the 43rd day of the experiments and prior to sacrificing, animals were fasted overnight. All rats were anesthetized by ketamine and xylazine (80 and 6 mg/kg, i.p, respectively).

After inducing deep anesthesia, abdominal cavity was opened. Blood collection was performed, and liver sections from left lateral lobe were rapidly removed, frozen in liquid nitrogen and then, stored at -80°C (Sellmann et al., 2015 ▶). Another liver tissue sample from left lateral lobe was fixed in formalin solution (10%).

**Serum biochemistry analysis**

 Collected blood samples were centrifuged (6000 g for 10 min) and stored at -80°C. The serum level of liver functional markers, aspartate aminotransferase (AST), alanine aminotransferase (ALT), and alkaline phosphatase (ALP), and the level of total cholesterol, triglyceride (TG), and high-density lipoprotein (HDL) were determined using an automatic serum Automatic analyzer (BT 1500-A-A, Rome, Italy) (Hussien and Shoman, 2013 ▶).Low-density lipoprotein-cholesterol (LDL-C) was calculated as total cholesterol – (HDL-C + triglyceride /5).

**Histopathological analysis of the liver**

Formaldehyde fixed liver tissue, were embedded in paraffin, and sectioned (5 µm) by using a microtome. The sections were stained by hematoxylin and eosin. Histopathological analysis was done blindly under a light microscope.

**Liver histology scoring**

The following grading method was used to determine the histopathological changes of the liver. Fatty change was graded according to the percentage of hepatocytes containing macro-vesicular fat (grade 1: 0–25%; grade 2: 26–50%; grade 3: 51–75%; and grade 4, 76–100%) (Ip et al., 2003). The degree of inflammation and accumulation of RBCs is expressed as the mean of 10 different fields within each slide that had been classified on a scale of 0–3 (0: normal; 1: mild; 2: moderate; and 3: severe) (Bruck et al., 2003 ▶).

**Assessment of the activity of antioxidants and level of lipid peroxidation**

The frozen liver tissue was homogenized in1ml phosphate buffered saline (pH 7.4) and then centrifuged (15000 rpm for 15 min). The TAC and MDA levels in homogenates of liver tissue, were measured using specific kits according to the manufacturer’s instructions.

**Measurement of the expression of miRs and mRNAs**

Total RNA (miRNAs and mRNAs) were extracted from the frozen serum samples using miRNeasy/Plasma kit and RNeasy plus mini kit, respectively (Qiagen, GmbH, Germany). After determination of the purity and concentration of the extracted RNA, cDNA was synthesized (Qiagen, GmbH, Germany). To quantify the expression levels of studied microRNAs (*122* and *34a*) and mRNAs (*NFκB*, *TNF*-*α*, *IL*-*6*, *Nrf2* and *HO1*), semi-quantitative real-time PCR (qRT-PCR) was performed. Table 1 lists the sequence of forward and reverse primers used for amplifying of each gene. No-template negative control (H_2_O) was routinely run in every PCR. 

The levels of miRNAs and mRNAs expression were respectively normalized against *RNU6* (as an internal control) and glyceraldehyde-3-phosphate dehydrogenase (*GAPDH*) and the fold change was calculated using the 2^–ΔΔCt^ formula.

**Statistical analysis**

Results are expressed as means±standard error of means (SEM). One-way analysis of variance (ANOVA) with LSD *post hoc* tests was used for identification of significant differences among the studied groups (IBM SPSS statistics 16). Kruskal–Wallis test was used to analyze histopathologic scoring. 

**Table 1:** Sequences of real time PCR primers

Primers	Forward	Revers
*NFκβ*	ACCCGAAACTCAACTTCTGT	TAACAGCTGGGGGAAAACT
*TNFα*	TGTGCCTCAGCCTCTTCTCATTC	CATTTGGGAACTTCTCCTCCTTG
*IL-6*	GGTCTTCTGGAGTTCCGTTT	AGTTGGGGTAGGAAGGACTA
*Nrf2*	CTCTCTGGAGACGGCCATGACT	CTGGGCTGGGGACAGTGGTAGT
*HO1*	TCAGCACTAGTTCATCCCAG	AAGCTTTCTTAGAGGCCCAA
*GAPDH*	TGCTGGTGCTGAGTATGTCGTG	CGGAGATGATGACCCTTTTGG

*NFκβ*: nuclear factor κβ, *IL-6*: interleukin6, *TNFα*: tumor necrosis factor α, *Nrf2*: nuclear factor erythroid- related factor 2, *HO1*: heme oxygenase-1, and *GAPDH*: glyceraldehyde-3-phosphate dehydrogenase.

## Results

**Analysis of the given dust**

**Figure 2:**
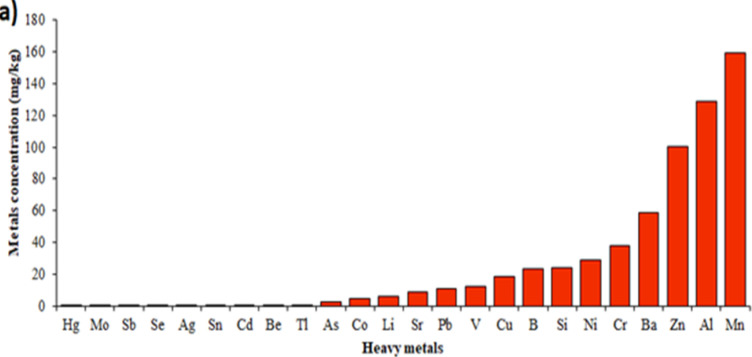
Mean content of the investigated heavy metals in collected dust

Analyzing the composition of the given dust in term of the heavy metals content showed the existence of 25 heavy metals: Ag, Al, As, B, Ba, Be, Cd, Co, Cr, Cu, Hg, Li, Mn, Mo, Ni, Pb, Sb, Se, Si, Sn, Sr, Ti, V and Zn. The concentrations of Aluminum, Manganese and Zinc in the given dust were the higher compared to blank sample (Figure 2). 

**Rat whole body exposure to dust accompanied by HFD, induced non-alcoholic fatty liver disease phenotype**

To elucidate *in vivo* effects of whole body exposure to dust, male Wistar rats under HFD, were exposed to dust or clean air (CA) after 42 days. We tried to create a situation similar to the real life by this exposure. During the exposure period, the mean concentration of PM10 and PM2.5 in the exposure chamber was respectively 1388 and 416.4 µg/m^3^ and it was 50 µg/m^3^ for CA group.

**Changes in macroscopic appearance and microscopic architecture of the liver following exposure to dust**

**Figure 3:**
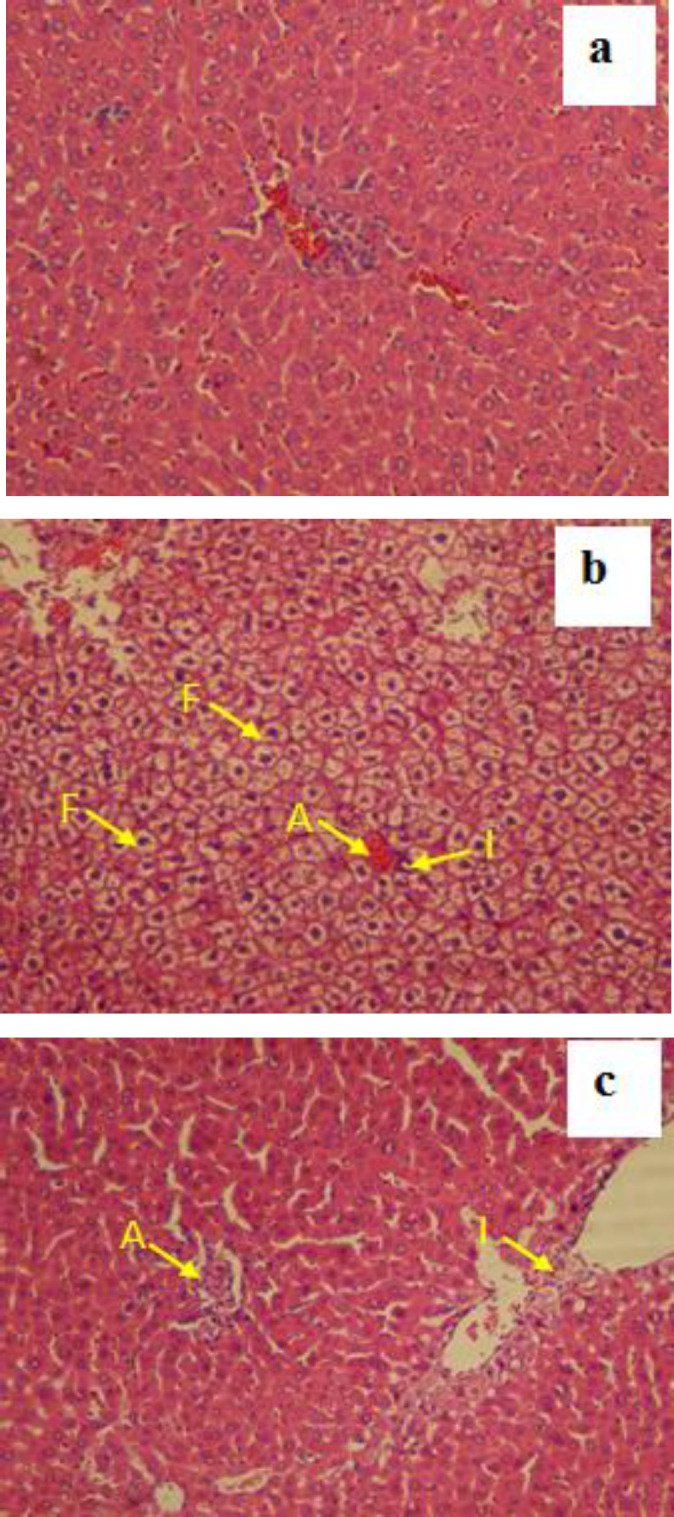
Representative microscopic images (magnification X250) of H&E-stained liver sections following dust and clean air (CA) exposure accompanied by high-fat diet (HFD). a: HFD+clean air group showed normal appearance and there were no histopathological changes; b: The liver in HFD+N/S+ Dust exposed rats showed inflammation (I), accumulation of RBCs (A) and fatty deposit (F); and c: Animals in HFD+gallic acid + Dust group showed mild inflammation and blood cell accumulation but no signs of fatty deposit

As shown in Figure 3, accumulation of RBC and fatty deposit was seen in HFD+N/S+Dust rats’ liver (Figure 3b). No sign of fatty deposit was seen in HFD+GA+Dust group, but mild inflammation and accumulation of RBCs were seen in this group. H&E staining revealed that inflammation, accumulation of RBCs and fatty deposit in HFD+N/S+Dust group, were significantly higher than HFD+CA group. GA pretreatment also significantly reduced these levels (Table 2).

**Table 2:** NAFLD grading and staging in Wistar rats exposed to clean or dusty air

**RBCs accumulation**	**Fatty deposit**	**Inflammation**	**Groups**
0.76±0.26	00±00	0.85±0.8	HFD+CA
1.58±0.29 *	3.6±8.6*	1.42±0.22*	HFD+N/S+dust
0.07±0.01*#	.46±.07*#	0.08±0.02*#	HFD+GA+Dust

*p<0.05compared to the HFD+CA group. #p<0.05 compared to the HFD+N/S+Dust group.

**Gallic acid increased total antioxidant capacity (TAC) while decreased hepatic malondealdehyde (MDA) level in dust-exposed rats under high-fat diet**

**Figure 4:**
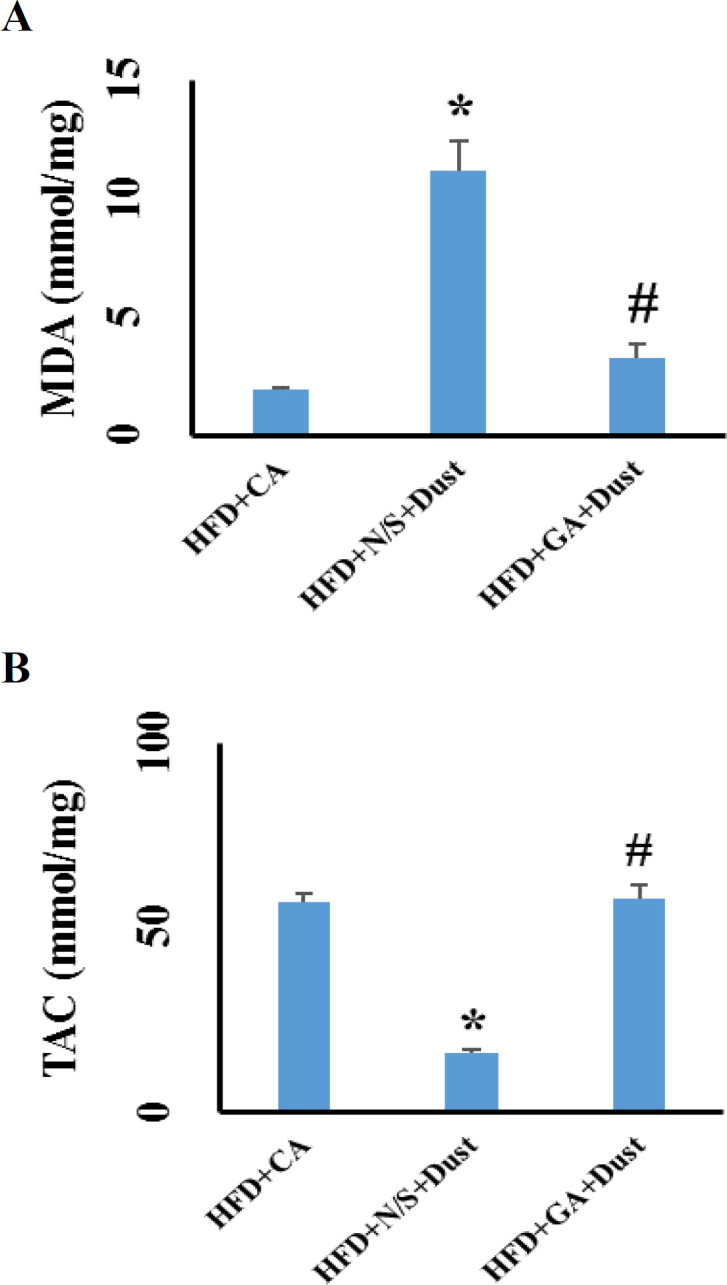
Gallic acid decreased hepatic malondialdehyde (MDA) level while increased hepatic level of total antioxidant capacity (TAC) in rats exposed to dust under high-fat diet (HFD). Data are expressed as the means±SEM.*p˂0.05 compared to the HFD+CA group and #p<0.05 compared to the HFD+N/S+Dust group

As indicated in Figure 4, dust exposure significantly decreased hepatic TAC level in the HFD+N/S+Dust rats compared to the HFD+CA group while increased hepatic MDA level (p˂0.05 in both cases). Pretreatment with GA at 100 mg/kg reverted TAC and MDA levels to those of the HFD+CA group.

**Gallic acid pretreatment decreased serum levels of miR-122 and miR-34 following dust exposure in rats under high-fat diet**

**Figure 5:**
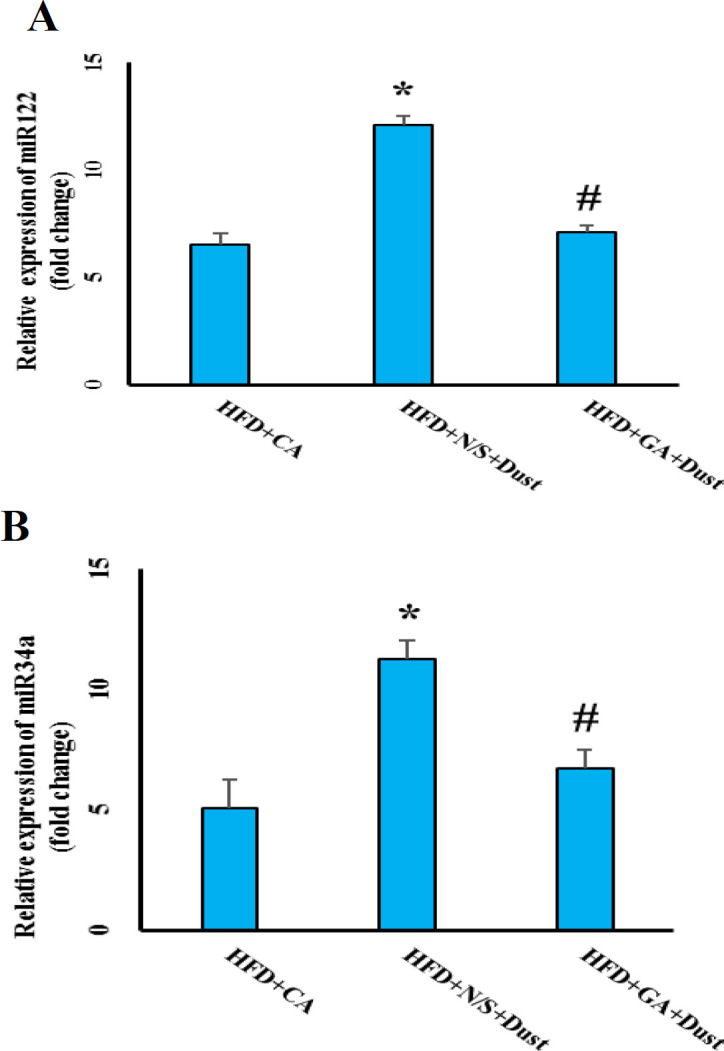
Effect of dust exposure and GA pretreatment on serum levels of (a) miR-122 and (b) miR-34a in rats under high-fat diet (HFD). The data are presented as the mean±SEM. *p<05 compared to the HFD+CA group; and #p<0.05 compared to the HFD+N/S+Dust group

As illustrated in Figures 5 (a and b), serum levels of miR-34a and miR-122 significantly increased following dust exposure in rats under high-fat diet (p˂0.05 in both cases). GA pretreatment (100 mg/kg) just before dust exposure, reverted the level of mir-34a and miR-122 to that of the HFD+CA group.

**Gallic acid improved lipid profile in dust-exposed rats under high-fat diet**

**Figure 6:**
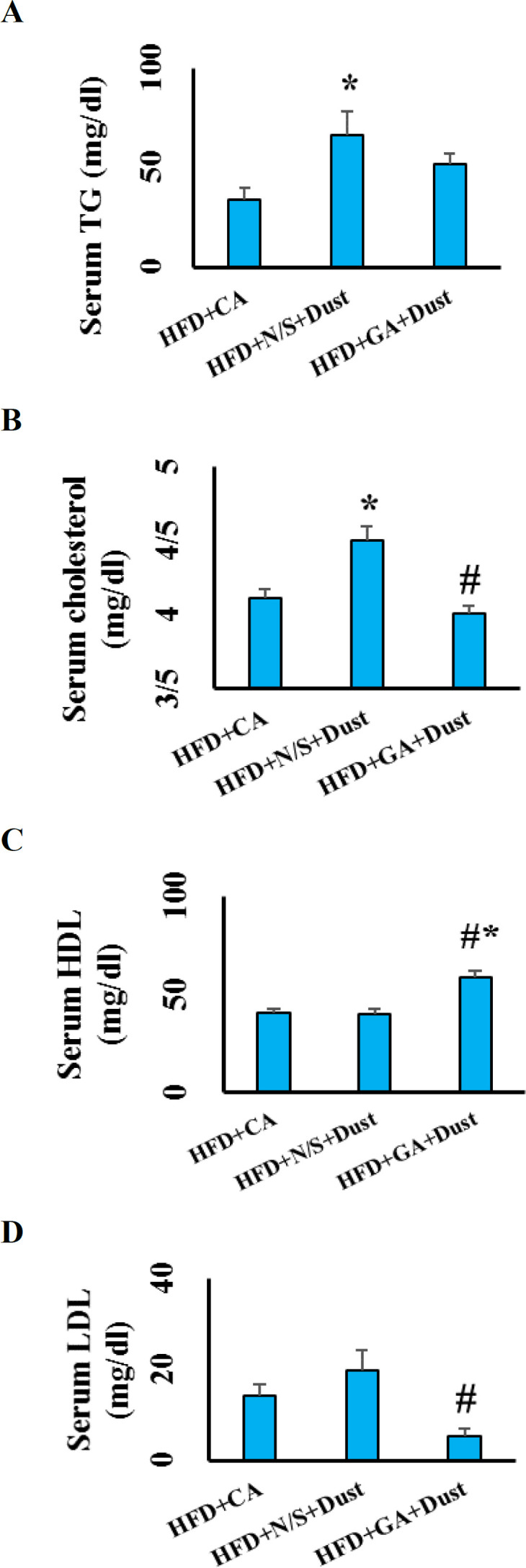
Gallic acid improved lipid profile in rats exposed to dust under high-fat diet (HFD). (a) Serum levels of triglyceride (TG); (b) Serum levels of cholesterol; (c); Serum levels of high-density lipoprotein (HDL) and (d) Serum levels of Low-density lipoprotein (LDL). The data are presented as the mean±SEM. *p˂0.05 compared to the HFD+clean air group, and #p<0.05 compared to the HFD+N/S+Dust group

Exposure to dust for 6 weeks, significantly increased serum levels of TG and cholesterol in the HFD+N/S+Dust rats compared with the HFD+CA group (p˂0.05 in both cases) (Figure 6a-b). 

There was no significant difference in serum LDL and HDL level between the HFD+CA and HFD+Dust groups. Pretreatment with GA at 100 mg/kg for 6 weeks just before dust exposure, significantly decreased serum level of TG, cholesterol and LDL, while increased serum HDL level (p˂0.05 in all cases).

**Gallic acid improved liver enzyme disturbances in dust-exposed rats under high-fat diet**

**Figure 7:**
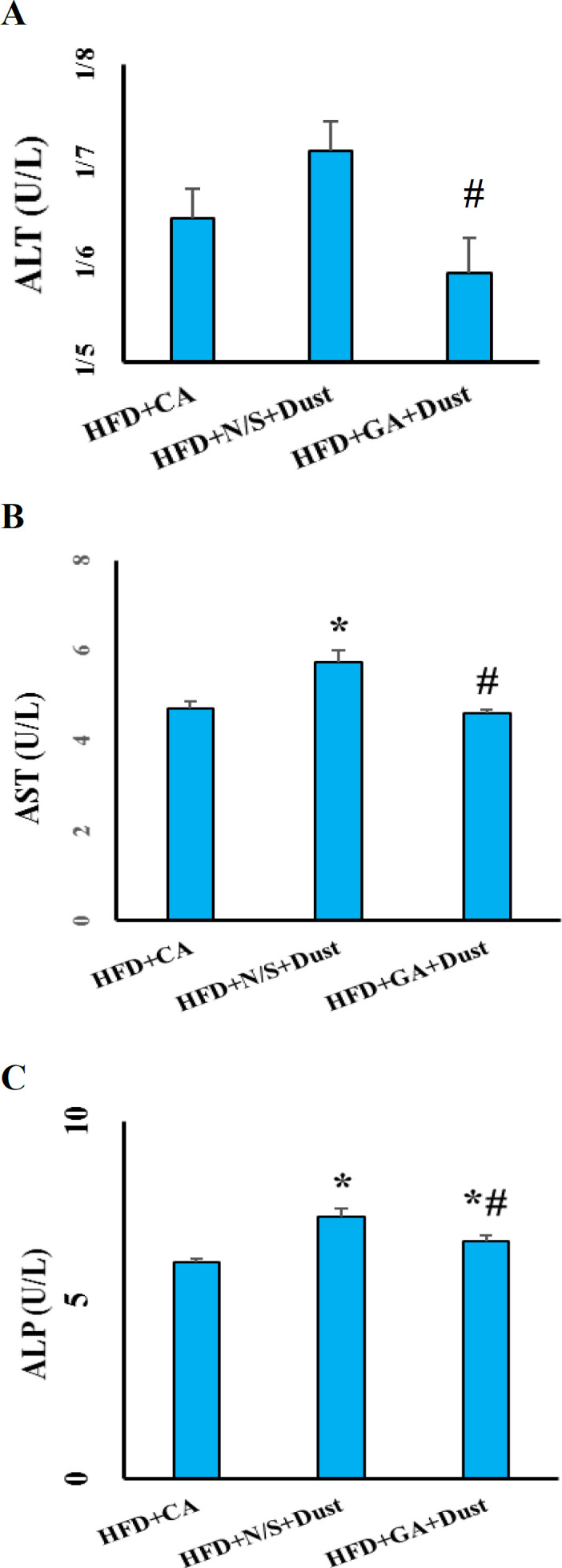
Gallic acid improved liver enzyme disturbances in rats exposed to dust under high fat diet (HFD). (a) Serum levels of alanine aminotransferase (ALT); (b) Serum levels of aspartate aminotransferase (AST); and (c) Serum levels of alkaline phosphatase (ALP). Data are presented as the mean±SEM. *p˂0.05 compared to the HFD+clean air group, #p<0.05 compared to the HFD+N/S+Dust group

As demonstrated in Figure 7, there was no significant difference in ALT serum level between CA- and Dust-exposed rats under HFD. Serum levels of AST and ALP increased significantly in the HFD+N/S+Dust group compared with the HFD+CA rats (p˂0.05 in both cases) (Figure 7a-c). GA pretreatment decreased serum levels of AST, ALT and ALP (p˂0.05 in all cases). 

**Effect of dust exposure and gallic acid pretreatment on mRNA expression of** ***NF-κβ*** **, pro- inflammatory cytokines,** ***Nrf2*** **and** ***HO1***

**Figure 8:**
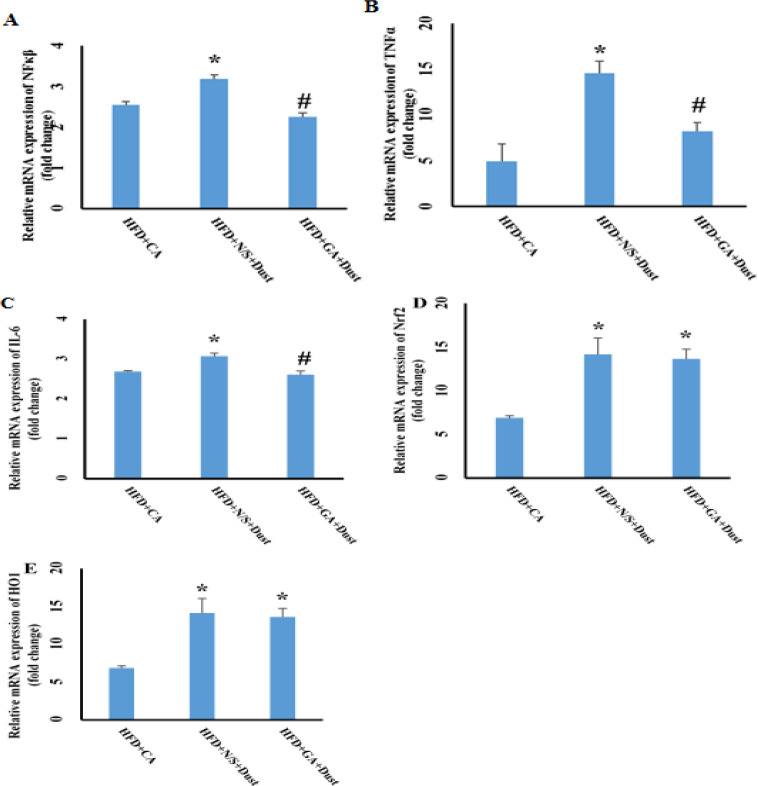
Effect of dust exposure and Gallic acid pretreatment on gene expressions of pro- inflammatory cytokines,*NF-κB*, *Nrf2*, *HO1* under high fat diet (HFD). *p<0.05compared to the HFD+clean air group; #p<0.05 compared to the HFD+N/S+Dust group

As shown in Figure 8(a-c), exposure to dust increased the mRNA expression levels of *NF-κβ*, *IL-6* and *TNF-α* in the HFD+N/S+Dust compared to the HFD+CA group (p˂0.05 in both cases). GA pretreatment before dust exposure significantly prevented the increment of these levels (p˂0.05 in both cases). Nrf2 and HO1 mRNA expression in the HFD+N/S+Dust and HFD+GA+Dust rats increased significantly (p˂0.05 in both cases). There was no significant difference between the HFD+N/S+Dust and HFD+GA+Dust rats in *Nrf2* and HO1mRNA expression.

**The origin of the collected dust**

**Figure 9:**
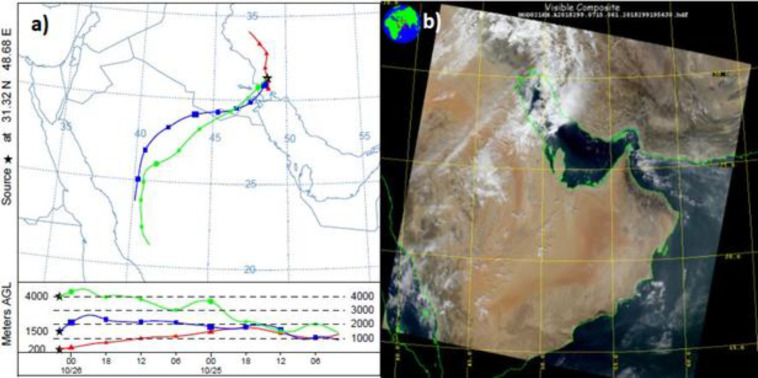
A backward HYSPLIT trajectory of the occurred dust in Ahvaz on October 26, 2018, b associated MODIS image over the region

One of several dust events that happened in autumn 2018 is demonstrated based on Hybrid Single Particle Lagrangian Integrated Trajectory (HYSPLIT) model, and MODIS data in Figure 9. The highest PM10 level of this event was725 µg/m^3^ at 05:00 AM on October 26, 2018 though there were other dust storms in the same period that reduced visibility in Ahvaz with more PM10 severity. PM10 concentration was greater than 2000 µg/m^3^. It appears that Saudi Arabia was the main source of the dust storm.

## Discussion

Dust composition is dependent on itsorigin. There is a direct correlation between dust composition(s) and its health side effects. Prior studies have shown dust components (PMs or heavy metals) when evaluated individually could endanger health status (Araujo, 2010 ▶; Cave et al., 2010 ▶; Hyder et al., 2013 ▶; VoPham, 2019 ▶). A human study has shown a direct correlation between serum levels of heavy metals and development of NAFLD features (VoPham, 2019 ▶). Our results showed that heavy metals concentration in the given dust washigher than normal levels. Higher concentrations of heavy metals in the given dust compared to the sample blank could serve as an important reason for functional and morphological changes observed inliver tissuse of the HFD+N/S+Dust rats. 

The results of the present study showed that whole body dust exposure accompanied by HFD, induced NAFLD features in rats. Our microscopic evaluations demonstrated that dust exposure accompanied by HFD, led to inflammation, accumulation of RBCs and fatty deposit in rats’liver compared to the HFD+CA group while gallic acid pretreatment significantly decreased inflammation, and accumulation of RBCs. However, in the liver of the HFD+GA+Dust rats, no signs of fatty deposit were seen. A previous study has shown protective effects of GA against HFD-induced hepatic steatosis (Chao et al., 2014 ▶). Generally, these findings showed HFD+Dust (alsoshown by the current results) and its components (as revealed by previous reports), caused liver damage, and GA pretreatment prevented emergence of NAFLD features. 

A previous study suggested that ulter fine particle (UFP) exposure triggers injury via oxidative stress induction (Brown et al., 2004 ▶). An earlier study has shown thatheavy metals are cable of oxidative stress induction (Ayres et al., 2008 ▶). Dust analysis showed that the amount of heavy metals was dramatically high. Thus, these findings showed that oxidative stress induction occurred due to high concentrations of heavy metals along with other components present in the given dust. However, increased levels of MDA related to dust exposure, could represent oxidative stress situation. A prior study has also reported that UFP exposure via systemic oxidative stress induction, increased MDA level (Delfino et al., 2011 ▶). In agreement with such data, the present study showed that liver MDA level increased in the HFD+N/S+Dust group compared to the HFD+CA rats. These findings showed that dust exposure accompanied by HFD, induced liver oxidative stress, increased lipid peroxidation, damaged hepatocytes, increased cell membranes permeability that ledto an increment in serum levels of ALT, AST, ALP and miR-122. This study showed that liver MDA level in the HFD+GA+Dust group was significantly lower than that of the HFD+N/S+Dust rats. A previous report has indicated the ROS scavenging activity and antioxidant-promoting activity of GA in ischemia/reperfusion liver injuries (Bayramoglu et al., 2015 ▶). Therefore, it can be concludedthat GA is able to inhibit oxidative stress.

There is a positive correlation between the serum levels of miR-34a and oxidative stress-induced injuries. Oxidative stress condition led to increased serum levels of miR-34a (Upton et al., 2012 ▶). The current study showed that serum levels of miR-34a and liver MDA level increased in the HFD+N/S+Dust rats. The present results also showed that GA pretreatment improved these levels. From these findings, we concluded that dust exposure accompanied by HFD, induced oxidative stress injury and GA pretreatment via reduction of oxidative stress- protected the liver. 

Liver structural damage and cellular leakage lead to the over release of liver amino transaminases and ALP from hepatocytes and elevated levels of these markers in serum (Jalili et al., 2015 ▶). The present results showed that dust exposure accompanied by HFD, increased serum levels of AST, ALT and ALP while GA pretreatment improved these levels. GA as an antioxidant inhibited lipid peroxidation, maintained cell membrane integrity and consequently, improved these levels.

Our results showed that serum level of TG and cholesterol increased in the HFD+N/S+Dust, while serum HDL and LDL were not significantly different between the HFD+CA and HFD+N/S+Dust groups. GA pretreatment improved lipid profile in dust-exposed rats under HFD. PMs exposure increased oxidative stress which in turn, adversely affected lipoproteins such as HDL. HDL modulates the plasma level of cholesterol (Araujo et al., 2008 ▶). Ambient air pollution increased adipose inflammation and insulin resistance (Sun et al., 2009 ▶). Insulin resistance increased lipolysis which in turn, increased plasma TG level. Then, our results in agreement with a previous study, have shown that air pollution disrupted lipid profile (Wei et al., 2016 ▶) while GA pretreatment via inhibiting oxidative stress and then, inhibiting lipid peroxidation improved lipid profile and prevented NAFLD development (Chao et al., 2014 ▶). 

This study showed that expressions of Nrf2 and HO1 increase in the HFD+N/S+Dust and HFD+GA+ Dust groups compared to the HFD+ CA group. A pervious study has shown that airborne particulate matter increases Nrf2 and HO1 mRNA expression (Araujo et al., 2008 ▶). In agreement with a previous report, our study has shown that air pollution and GA pretreatment increased mRNA expression of Nrf2 and HO1 (Yeh and Yen, 2006 ▶).

Oxidative stress activated NF-κβ pathway and increased expression of pro-inflammatory factors TNF-α, IL-1, and IL-6 (Sivandzade et al., 2019 ▶). The present results showed that dust exposure accompanied by HFD, increased the mRNA levels of NF-κβ, TNF-α, and IL-6. Similar findings were also reported following exposure to PMs (Vignal et al., 2017 ▶). Therefore, taken these results together, it is concluded that exposure to dust accompanied by HFD as shownhere or its components (PMs) as reported by previous studies, by activating the inflammatory pathway, damaged the liver and induced hepatic inflammation. 

Our findings showed that dust exposure caused a significant decrease in the liver TAC while GA pretreatment significantly increased TAC in rats under HFD. Air pollution components increased oxidative stress, disturbed oxidant-antioxidant balance and oxidant mediators conquered antioxidant defense. This situation makes organs susceptible to oxidative stress damages. A previous study has shown that air pollution exposure reduced both serum and organs antioxidant levels (Liu and Meng, 2005 ▶). Another study also showed that GA improved antioxidant capacity and exhibited protective effect on liver oxidative stress (Ahmadvand et al., 2017 ▶). Our findings were consistent with prior studies showing that dust exposure decreased antioxidant potency while GA pretreatment increased TAC and improved antioxidant capacity.

In conclusion, this study showed that HFD given for six weeks and exposure to dust, induced NAFLD in Wistar rats through inducing oxidative stress. Oxidative stress through activating the inflammatory pathways caused NAFLD features. Gallic acid pretreatment by inhibiting oxidative stress effectively protected liver function against HFD+Dust induced inflammation. 
